# Association between the First Occurrence of Asthma and Residential Greenness in Children and Teenagers in Taiwan

**DOI:** 10.3390/ijerph16122076

**Published:** 2019-06-12

**Authors:** Chia-Jung Hsieh, Pei-Ying Yu, Chun-Ju Tai, Rong-Hwa Jan, Tzai-Hung Wen, Shyang-Woei Lin, Chun-Chieh Tseng

**Affiliations:** 1 Department and Graduate Institute of Public Health, Tzu Chi University, Hualien 97004, Taiwan; cjhsieh@mail.tcu.edu.tw (C.-J.H.); 102324106@gms.tcu.edu.tw (P.-Y.Y.); 102313122@gms.tcu.edu.tw (C.-J.T.); 2 Institute of Medical Science, Tzu Chi University, Hualien 97004, Taiwan; janronghwa@yahoo.com.tw; 3 Department of Pediatrics, Tzu Chi Hospital Hualien, Hualien 970, Taiwan; 4 Department of Geography, National Taiwan University, Taipei 10617, Taiwan; wenthung@ntu.edu.tw; 5 Department of Natural Resources and Environmental Studies, National Dong Hwa University, Hualien 97401, Taiwan; shine@gms.ndhu.edu.tw

**Keywords:** asthma, preschool children, greenness space, air pollution

## Abstract

Green spaces have benefits but may also increase the risk of allergic disease. This study examined the association between the first occurrence of asthma and greenness exposure in children and teenagers. We conducted a 1:1 matched case-control study matched by sex, age, and the first diagnosis year with 7040 eligible subjects from a systematic sampling cohort database in Taiwan from 2001 to 2013. A normalized difference vegetation index (NDVI) value ≥0.4 was used as the criterion to determine the green space. The green cover images were then transformed to the green coverage rate in the township surrounding the residential areas of the asthma and control subjects. Conditional logistic regression analyses demonstrated that a significantly increased risk of asthma in preschool children was associated with the surrounding greenness after adjusting for urbanization level, frequency of healthcare provider visits, mean township family income, CO, NO_x_, and PM_2.5_. The risk of asthma occurrence increased significantly with increasing greenness exposure (*p*-trend < 0.05). Nevertheless, exposure to the highest greenness levels (81–100%) was not associated with a significantly higher risk of asthma occurrence than was exposure to the lowest values (0–20%) of greenness. This study suggests that green space design should consider more effective methods of reducing the allergy impact.

## 1. Introduction

Asthma is the most prevalent childhood chronic airway disease. In Taiwan, the prevalence of asthma is 5.1% in the general population; however, the prevalence rate can be twice that percentage or even higher in children [1]. The main etiology of asthma in children is not fully understood and still needs clarification. With regard to environmental risk factors, a variety of allergens such as pollens, molds, and dust mites can trigger asthma [2]. In addition, several studies have indicated that the increasing asthma prevalence is associated with urban air pollutants, especially long-term exposure to carbon monoxide (CO) [3], nitrogen oxides (NO_x_) [3,4], and particulate matter 2.5 (PM_2.5_) [5].

Recently, there has been substantial interest in the relationship between asthma and the amount of residential greenness. Green environments are often associated with benefits such as reducing heat, noise, and air pollution exposure [6]; however, exposure to greenness may also induce some health risks [7,8,9]. At present, the results from epidemiological studies investigating the linkage between residential greenness and asthma are both limited and inconsistent. Some studies reported that exposure to greenness may increase the risk of asthma [9,10], but others reached either the opposite conclusion [11,12] or found no association [13]. The inconsistencies among these different studies may be related to their study designs, populations, definitions of asthma, and greenness measurement methods.

Although many studies have investigated the relationships between asthma and residential greenness, some studied only specific age ranges. Relatively few data exist for age groups younger than teenagers. In addition, while some important air pollution contaminates such as CO, NO_2_, and PM_2.5_ are significantly related to asthma, few studies have adjusted for these factors—possibly because there were no air monitoring sites located near the study subjects’ residences. Consequently, in this study, we included all age groups younger than teenagers using Taiwan’s National Health Insurance Data. The advantage of this data set is that all the asthma subjects were diagnosed by doctors. The association between the first occurrence of asthma and the amount of residential greenness exposure was determined after adjusting for a rural/urban indicator and for CO, NO_2_, and PM_2.5_ concentrations.

## 2. Materials and Methods

### 2.1. Subject Inclusion

**Figure 1:**
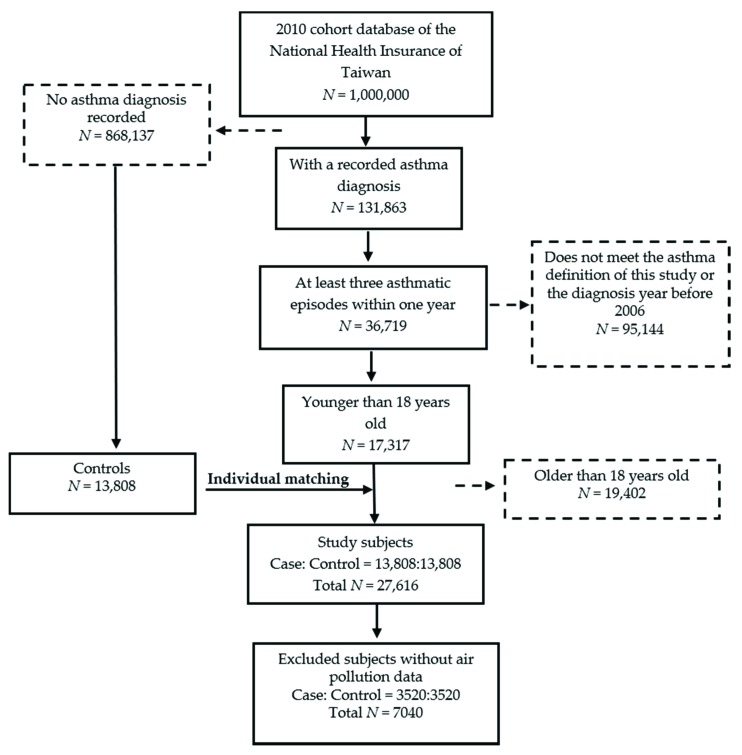
A flowchart of participant inclusion in this study.

We systematically sampled the cohort database of 1,000,000 insured individuals from the National Health Insurance of Taiwan from 2001 to 2013 (Figure 1) and conducted a 1:1 matched case-control study, matched by sex, age, and first diagnosis year.

The diagnoses were made according to the International Classification of Diseases, 9th Revision, Clinical Modification (ICD-9-CM), and codes 493.00, 493.90, and 493.91 were used to determine the presence of asthma. ICD-9-CM is a list of codes used to represent doctor’s diagnoses and to classify clinical diseases. In this study, we focused only on subjects younger than 18 years who had experienced at least three asthmatic episodes within one year. Consequently, the current study included 17,317 individuals with asthma who were first diagnosed with asthma between 2006 and 2013. Then, control subjects who had not been diagnosed with asthma were individually matched with the subjects with asthma according to sex, age, and first diagnosis year. Asthma and control subjects without available air pollution monitoring data or sufficient information were removed from the study. Finally, the total number of study subjects was 7040, including 3520 asthmatic subjects and 3520 control subjects. The study was approved by the Research Ethics Committee of Tzu Chi General Hospital (No: IRB104-130-C, approved on 19 November 2015).

### 2.2. Greenness Exposure

We used the normalized difference vegetation index (NDVI) to measure the greenness surrounding the residential areas. In the current study, a value of NDVI ≥ 0.4 was adopted as the criterion for determining green space. The green cover was obtained from images acquired by Landsat Thematic Mapper (TM), Enhanced Thematic Mapper Plus (ETM+), and Thermal Infrared Sensor (TIRS) satellites and had an overall accuracy of 92.8% (*p* < 0.05) [14]. Interactive data language (IDL), a programming language used to analyze image data, was employed for the image computations. For each study subject, the green cover images and data were transformed to the green coverage rate in the township surrounding the residential areas of the asthma and control subjects. To investigate the relationship between greenness exposure and asthma incidence, we differentiated the greenness exposure range into the following five exposure levels: 0–20%, 21–40%, 41–60%, 61–80%, and 81–100%. The green coverage map in Taiwan was drawn by ArcGIS 9.3.1. (ESRI Inc., Redlands, CA, USA). In Taiwan, the basic administrative unit is the township. Townships include an average of 55,000 individuals and 20,000 households. Among all the townships in Taiwan, the highest population density is in the western coastal urban area, which has more than 20,000 persons per km^2^ but has a green cover of only 3%. The lowest population density is in the eastern mountainous area, with only 8 persons per km^2^ but a green cover of 97% [15].

### 2.3. Air Pollution Monitoring Data

Air pollution monitoring sites sharing the same township codes with the case or control subjects were used as the sources of air pollution data. No township contained two or more air pollution monitors. Subjects with no air pollution monitor available in their resident townships were not included in this study. Because our cohort database consists of secondary data from which personal identification was removed, we defined the subjects’ residential townships based on the areas where insured peoples’ respiratory infections (ICD-9-CM: 460–466, 480–487) were treated by local clinics [16]. The daily and monthly average concentrations of three important air pollutants related to asthma, CO, NO_2_, and PM_2.5_, were available for download from the Taiwan Air Quality Network (http://taqm.epa.gov.tw/taqm/en/default.aspx). To match the study period, we collected air pollution data from 2005 to 2012.

### 2.4. Data Management and Analysis

Our data were divided by age to clarify the effects on asthma incidence. Although the cohort database does not contain personal identification, we were able to infer the subjects’ residential townships by following a method outlined in a previous study [14]. Information concerning the subjects’ township residence was helpful in linking asthma data with air pollution data and the greenness exposure level. Based on the year in which a subject’s asthma was newly diagnosed, we linked the asthma incidence to the green coverage rate and to air pollutant concentrations in the year prior to the onset of asthma. The air pollutant concentrations and greenness exposure levels were used as the annual average values. In addition, we conducted a conditional logistic regression analysis to calculate the odds ratios (ORs) for asthma occurrence and greenness exposure levels. Four important confounders, including air pollutants, urbanization degree, frequency of healthcare provider visits, and mean township family income, were used for adjustment. The degrees of urbanization were based on a previous study [17]; Taiwan was divided into four different urbanization degrees according to the development level of each administrative region. The statistical analyses were all performed with SAS 9.4 (SAS Institute, Inc., Cary, NC, USA).

## 3. Results

### 3.1. Green Coverage Change in Taiwan in the Past Decade

**Figure 2:**
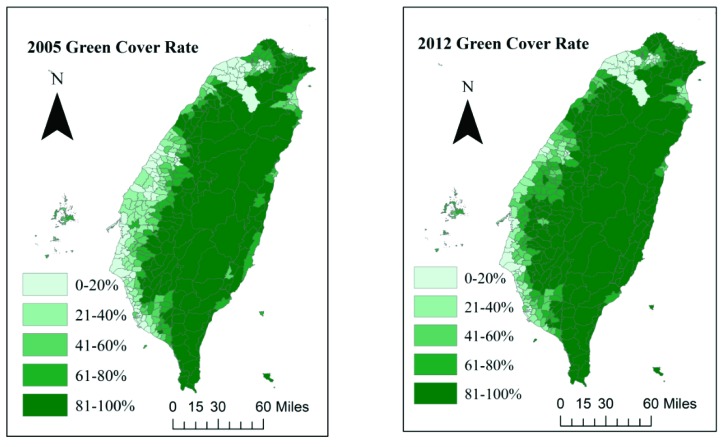
Green coverage changes in Taiwan over the eight study years.

Figure 2 shows the change in green coverage in Taiwan between 2005 and 2012. Because the greenness data were collected from 2005 to 2012, we demonstrated the changes in greenness distribution in Taiwan after eight years. Hualien County and Taitung County in the eastern region are less developed than the western regions and retain the most green coverage. The green coverage level of eastern Taiwan is 80–100%, which is the highest level in this study. The vegetation cover in the western region is not as extensive as that in the east. The western and southwestern regions in Taiwan tend to have lower average green coverage rates due to the many industrial and highly urbanized areas. Consequently, a Spearman correlation analysis showed a negative correlation between the greenness coverage and air pollutant concentrations for CO (*r* = −0.59, *p* < 0.0001), NO_2_ (*r* = −0.61, *p* < 0.0001), and PM_2.5_ (*r* = −0.26, *p* < 0.0001).

### 3.2. Characteristics of Asthma Cases and Controls

The descriptive statistics for asthma cases and controls are listed in Table 1. Because the age and gender of asthma cases and controls were already matched, there was no significant difference between these two groups. However, the distributions of greenness exposure, urbanization, frequency of healthcare provider visits, and mean family income between the asthma cases and the controls were significantly different. Because our database did not contain personal information, some important individual confounders were unavailable. Consequently, the factors that demonstrated significant differences between the study cases and controls in Table 1 needed to be adjusted because these factors may represent health-related factors at the individual level.

### 3.3. The Relationship Between Greenness Exposure and Asthma Incidence Before the Age of 18

The results presented in Table 2 were derived from two different models that examined the relationship between green cover exposure and asthma incidence. Because the concentrations of the air pollutants CO and NO_2_ were strongly correlated (*r* > 0.7), we had to adjust those two variables independently in models 1 and 2. Table 2 also shows that the risk of asthma occurrence was significantly higher in subjects with a higher greenness exposure levels than in those with lower greenness exposure levels, and a dose-dependent response was observed (*p*-trend = 0.0393 and 0.0289, respectively). The risk of asthma occurrence in the subjects exposed to 21% to 80% greenness coverage was 1.17–1.34 times higher than that in the subjects exposed to the lowest (0–20%) greenness level (*p* < 0.05). However, the difference in the risk of asthma occurrence between the subjects exposed to the highest greenness level (81–100%) and those exposed to the lowest greenness levels was not significantly different (*p* = 0.3674 and 0.3142). Table 2 demonstrates that the risk of asthma occurrence in males and females increased as the level of greenness exposure increased except at the highest greenness exposure level (81–100%). There was only a small difference in ORs between males and females when they were exposed to greenness. Exposure to 21–40% greenness resulted in a significant asthma risk only in males.

### 3.4. The Relationship Between Greenness Exposure and Asthma Incidence in Preschool Children

Table 3 shows results similar to those in Table 2, demonstrating that the risk of asthma occurrence increased significantly in preschool children (0–5 years old) as the level of greenness exposure increased. However, no dose-dependent response trend was found in either model 1 or 2. This resulted in significant ORs for asthma occurrence when comparing higher (21–80%) greenness exposure levels with the lowest level (0–20%). Similarly, when the greenness exposure level was the highest (81–100%), the exposure level did not significantly affect asthma occurrence.

### 3.5. The Relationship Between Greenness Exposure and Asthma Incidence in Subjects 6 to 17 Years Old

Table 4 presents similar results to those presented in Table 2 and Table 3 although no dose-dependent response was observed (*p*-trend = 0.1309 and 0.1442, respectively). In addition, the risk of asthma occurrence in school-age subjects exposed to 61% to 80% greenness coverage was the highest, and the difference was significant (*p* = 0.0344 and 0.0332). Similarly, the risk for subjects exposed to the highest greenness level (81–100%) was not significantly different from the risk for those exposed to the lowest greenness levels (*p* = 0.3963 and 0.4315). Consequently, the ORs across the NDVI categories for both younger and older children were very similar.

## 4. Discussion

Over the past eight years, due to policies promoting tree-planting activities, carbon reduction projects, and green building regulations to support sustainable development, the green area in Taiwan has gradually increased. In 2012, the overall green coverage increased to 1.2 times the green coverage in 2005. Policy promotes the expansion of a green environment mainly because such environments not only improve physical health but also reduce psychological stress [7]. In addition, increasing green space may reduce the impact of global warming [18]. However, the question of whether green environments provide environments for certain allergens such as fungi, spores, or pollen that induce allergic diseases has attracted public attention.

At present, the results of investigations into the association between greenness exposure and asthma in children are still inconsistent [7,9,10,19]. Our current results may partially explain the inconsistent results of the available research. Although the risk across the NDVI categories for younger and older children were very similar, some significant effects were only observed in preschool children. The subjects in previous investigations were mostly older than six years. The susceptible age groups in our study (age 0–5) are similar to those in the study by Andrusaityte et al. (ages 4–6), and these two studies obtained similar results: a slightly increased risk of asthma in subjects associated with high levels of surrounding greenness [10]. These findings may be related to the relatively longer time that preschool children stay at home; thus, green coverage around their place of residence has a greater impact on them in terms of asthma occurrence. Children over six years old and adolescents attend school; therefore, they spend less time at home. Consequently, the green coverage rate surrounding their place of residence may have less of an impact on them in terms of asthma occurrence. Although the age groups in one Canadian study were also similar to those in our study, a difference in the way the studies define greenness exposure makes it difficult to compare the results directly. The greenness index values in the Canadian study were obtained for the period during gestation [11].

In addition to the age effect, another interesting finding is that the highest green coverage level (81–100%) was not significantly correlated with the risk of asthma occurrence. Although we did not find that exposure to the highest level of green coverage had a protective effect against asthma occurrence, it is very likely that a high green coverage level confers some health benefits that offset the potential risk of asthma occurrence due to greenness exposure. For example, the highest level of greenness cover may reduce psychophysiological stress and may increase physical activity [10]. In addition, because of its high degree of biodiversity, a high green coverage may increase human immune function, which can positively impact human microbiota and the activity of macrophage cells [13,20,21]. Children with better immune function may have a reduced risk of inflammatory and allergic conditions [22]. Our results also indicated that the asthma risk associated were quite similar for males and females. However, the literature indicates that asthma and wheezing are more prevalent in males than in females in the younger age groups [23]. The detailed mechanism is still unknown but may be related to immunological and hormonal factors [24].

The inconsistent results from previous studies demonstrate that the effect of greenness exposure on asthma occurrence is complex and needs to be further investigated. Our findings also suggest that the effect of green coverage on asthma occurrence is neither absolutely positive nor negative. In fact, exposure to green coverage may have a “threshold effect” on asthma occurrence. The compositions of areas with high levels of green coverage may be different from those with low levels of green coverage. Areas with higher levels of green coverage may include forests and natural green spaces. However, areas with lower levels of green coverage may include parks, urban green spaces, or artificial landscaping. Compared with parks, urban green spaces, and landscaping, forests and parks contain different flora and substances with varied allergenicity [25]. Exposure to different allergens results in different asthma risks. This outcome may explain why no significant increase in asthma risk occurs when subjects are exposed to the highest level of green coverage. This finding also indicates that authorities should not simply focus on increasing green coverage. A healthy green area should be carefully planned, with efforts made to maintain biodiversity, to carefully control exotic species, and to select for plant species that produce few allergens [25].

The current study faced two limitations. First, we used an identification-free database and thus could not adjust for other personal asthma-related risk factors. Because of the lack of personal information, we were also unable to identify where the patients actually lived. Most previous studies either considered the greenness exposure period starting prenatally (i.e., during pregnancy) or at the time of birth or of the interview [7,8,9,10,11,12,13]. Consequently, we used the current greenness exposure (i.e., in the previous 12 months) to explore our hypotheses due to a lack of information regarding the participants’ families’ residential addresses during pregnancy and lifetime migration records. To avoid biases resulting from the misuse of residential addresses, lifetime exposure was difficult to determine in this study. Because of the lack of residential information, we tried to determine the subjects’ residential townships by following the method reported the study by Lin et al., which referenced the local clinic locations where insured people receive treatment for respiratory infections [16]. The correlation coefficient obtained through this method can predict a subject’s residential township in urban and rural areas with an accuracy greater than 0.9 [16]. In addition, we also examined the data from the period 6 to 12 months prior to diagnosis/inclusion in this study and found that 86% (6064/7040) of the cases and controls lived in the same residential area during the 12 months prior to inclusion. This result indicated that the residential mobility in our study might be low. Nevertheless, spatial autocorrelation may still occur between neighboring townships in our study. Finally, although we adjusted for several factors in the models, some important risk factors for asthma at the individual level could not be included in the analyses, which means that the relationship between greenness exposure and asthma still requires further investigation. Second, we could not identify the primary plant species at each level of green coverage; thus, any correlation between the green coverage area and plant species still needs to be clarified.

An advantage of our study was the inclusion of a relatively large number of subjects. In addition, we used a definition of asthma based on medical records: Each subject with asthma had experienced at least three asthmatic episodes within one year. This approach is more rigorous than determining the presence of asthma based on a questionnaire. Another advantage is that this study also considered the impact of outdoor air pollutants on asthma. Because exposure to green coverage comes from the outdoor environment, outdoor air pollution should also be considered in such analyses. Outdoor air pollutants are factors that affect asthma but have less commonly been discussed in other related studies. Further investigation may still be needed to evaluate the associations among the green space composition, air pollutants, and personal risk factors related to asthma.

## 5. Conclusions

This study demonstrates that the risk of a first asthma occurrence is significantly associated with higher levels of greenness exposure in preschool children. Nevertheless, exposure to green coverage may have a threshold effect on asthma occurrence. Exposure to the highest level of green coverage did not lead to a significantly increased risk of asthma. These results indicate that the effect of greenness exposure on asthma is quite complex even though we adjusted for some important air pollutants related to asthma occurrence in the models. The highest level of green coverage not only has the largest area but also may contain a relatively higher level of biodiversity, which may have a positive impact on the human immune system, offsetting any potentially negative impact on asthma incidence. Green spaces are associated with both health benefits and allergic risks. The results of the current study indicate the importance of designing green spaces and their flora to balance the health benefits and risks of exposure to greenness.

## Figures and Tables

**Table 1:** Characteristics of the asthma cases and controls.

Characteristics	Non-Asthma (*N* = 3520)	Asthma (*N* = 3520)	*p*-Value
	*N* (%)		
Age			1.0000
0–5	2531 (71.9)	2531 (71.9)	
6–18	989 (28.1)	989 (28.1)	
Gender			1.0000
Male	2007 (57.0)	2007 (57.0)	
Female	1513 (43.0)	1513 (43.0)	
Greenness			0.0367
0–20%	855 (24.3)	842 (23.9)	
21–40%	961 (27.3)	1031 (29.3)	
41–60%	815 (23.2)	745 (21.2)	
61–80%	555 (15.8)	606 (17.2)	
81–100%	334 (9.5)	296 (8.4)	
Urbanization			<0.0001
1 (Most urbanized)	738 (21.0)	1030 (29.3)	
2	2359 (67.0)	2228 (63.3)	
3	346 (9.8)	225 (6.4)	
4 (Least urbanized)	77 (2.2)	37 (1.0)	
Frequency of visits to healthcare providers			<0.0001
≤15 times/year	1558 (44.3)	668 (19.0)	
16–44 times/year	1274 (36.2)	1350 (38.4)	
45–60 times/year	502 (14.2)	968 (27.5)	
>60 times/year	186 (5.3)	534 (15.1)	
Mean family income of townships			<0.0001
<794,000 NTD	1020 (29.0)	772 (21.9)	
794,000–862,000 NTD	935 (26.6)	802 (22.8)	
862,000–989,000 NTD	637 (18.1)	774 (22.0)	
>989,000 NTD	928 (26.4)	1172 (33.3)	

NTD, New Taiwan Dollar.

**Table 2:** Odds ratios (OR) and 95% confidence intervals (CIs) for asthma occurrence according to the level of greenness exposure before the age of 18.

Greenness Exposure	Model 1		Model 2	
	OR (95% CI)	*p*-Value	OR (95% CI)	*p*-Value
0–20%	Reference		Reference	
21–40%	1.17 (1.06–1.30)	0.0027	1.17 (1.06–1.30)	0.0027
41–60%	1.28 (1.12–1.46)	0.0003	1.28 (1.12–1.46)	0.0002
61–80%	1.32 (1.13–1.53)	0.0003	1.34 (1.15–1.56)	0.0002
81–100%	1.09 (0.91–1.31)	0.3674	1.10 (0.92–1.32)	0.3142
*p* for trend		0.0393		0.0289
Males				
0–20%	Reference		Reference	
21–40%	1.19 (1.04–1.37)	0.0128	1.19 (1.04–1.37)	0.0137
41–60%	1.27 (1.06–1.51)	0.0081	1.26 (1.06–1.50)	0.0085
61–80%	1.35 (1.10–1.64)	0.0032	1.35 (1.11–1.65)	0.0029
81–100%	1.12 (0.88–1.43)	0.3563	1.12 (0.88–1.43)	0.3439
*p* for trend		0.0742		0.0750
Females				
0–20%	Reference		Reference	
21–40%	1.14 (0.97–1.34)	0.1021	1.15 (0.98–1.35)	0.0936
41–60%	1.28 (1.05–1.57)	0.0156	1.30 (1.06–1.58)	0.0102
61–80%	1.28 (1.02–1.62)	0.0371	1.32 (1.04–1.67)	0.0213
81–100%	1.05 (0.79–1.39)	0.7491	1.07 (0.81–1.41)	0.6433
*p* for trend		0.2702		0.1915

Model 1 was adjusted for urbanization level, frequency of healthcare provider visits, mean township family income, CO, and PM_2.5_. Model 2 was adjusted for urbanization level, frequency of healthcare providers visits, mean township family income, NO_2_, and PM_2.5_. The OR and 95% CI values were estimated using a conditional logistic regression model. The *p*-trends were calculated using the continuous scale of the level of greenness exposure in the corresponding models.

**Table 3:** Odds ratios (OR) and 95% confidence intervals (CIs) for asthma occurrence according to the level of greenness exposure in preschool children (0–5 years old).

Greenness Exposure	Model 1		Model 2	
	OR (95% CI)	*p*-Value	OR (95% CI)	*p*-Value
0–20%	Reference		Reference	
21–40%	1.18 (1.04–1.33)	0.0106	1.18 (1.04–1.33)	0.0107
41–60%	1.28 (1.10–1.50)	0.0017	1.29 (1.11–1.50)	0.0012
61–80%	1.31 (1.10–1.57)	0.0031	1.33 (1.11–1.60)	0.0017
81–100%	1.07 (0.86–1.33)	0.5464	1.09 (0.88–1.34)	0.4470
*p* for trend		0.1180		0.0828

Model 1 was adjusted for urbanization level, frequency of healthcare provider visits, mean township family income, CO, and PM_2.5_; Model 2 was adjusted for urbanization level, frequency of healthcare provider visits, mean township family income, NO_2_, and PM_2.5_. The OR and 95% CI values were estimated using a conditional logistic regression model. The *p*-trends were calculated using the continuous scale of the level of greenness exposure in the corresponding models.

**Table 4:** The odds ratios (OR) and 95% confidence intervals (CIs) of asthma occurrence according to the level of greenness exposure in subjects from 6 to 17 years old.

Greenness Exposure	Model 1		Model 2	
	OR (95% CI)	*p*-Value	OR (95% CI)	*p*-Value
0–20%	Reference		Reference	
21–40%	1.17 (0.96–1.43)	0.1226	1.17 (0.96–1.43)	0.1314
41–60%	1.25 (0.97–1.62)	0.0840	1.24 (0.96–1.60)	0.0946
61–80%	1.36 (1.02–1.81)	0.0344	1.36 (1.03–1.81)	0.0332
81–100%	1.17 (0.82–1.67)	0.3963	1.15 (0.81–1.64)	0.4315
*p* for trend		0.1309		0.1442

Model 1 was adjusted for urbanization level, frequency of healthcare provider visits, mean township family income, CO, and PM_2.5_. Model 2 was adjusted for urbanization level, frequency of healthcare provider visits, mean township family income, NO_2_, and PM_2.5_. OR and 95% CI values were estimated using a conditional logistic regression model. The *p*-trends were calculated using the continuous scale of the level of greenness exposure in the corresponding models.
